# Reward Influences Masked Free-Choice Priming

**DOI:** 10.3389/fpsyg.2020.576430

**Published:** 2020-11-30

**Authors:** Seema Prasad, Ramesh Kumar Mishra

**Affiliations:** Center for Neural and Cognitive Sciences, University of Hyderabad, Hyderabad, India

**Keywords:** reward, masked priming, free-choice, attention, saliency

## Abstract

While it is known that reward induces attentional prioritization, it is not clear what effect reward-learning has when associated with stimuli that are not fully perceived. The masked priming paradigm has been extensively used to investigate the indirect impact of brief stimuli on response behavior. Interestingly, the effect of masked primes is observed even when participants choose their responses freely. While classical theories assume this process to be automatic, recent studies have provided evidence for attentional modulations of masked priming effects. Most such studies have manipulated bottom-up or top-down modes of attentional selection, but the role of “newer” forms of attentional control such as reward-learning and selection history remains unclear. In two experiments, with number and arrow primes, we examined whether reward-mediated attentional selection modulates masked priming when responses are chosen freely. In both experiments, we observed that primes associated with high-reward lead to enhanced free-choice priming compared to primes associated with no-reward. The effect was seen on both proportion of choices and response times, and was more evident in the faster responses. In the slower responses, the effect was diminished. Our study adds to the growing literature showing the susceptibility of masked priming to factors related to attention and executive control.

## Introduction

Masked primes influence behavior on a range of simple tasks (Marcel, 1983, but see Greenwald et al., 1996; Dehaene et al., 1998, 2006; Kouider and Dehaene, 2007; Newell and Shanks, 2014). Is it possible that we are more influenced by such masked information when they are important or valuable to us in some way? Since human perceivers attach value to external stimuli, it is likely that such additional reward information may induce greater priming effects. But, traditional theories of cognition suggest that unconscious processes are prototypical examples of automatic processes which are not prone to interference from other processes (Posner and Snyder, 1975). This has been taken to imply that only conscious processes are susceptible to any form of strategic control and nearly-invisible stimuli are outside the domain of cognitive control. But more recently, these views have changed and paved the way for more refined theories which allow for executive control over unconscious processing (Kiefer, 2012; Ansorge et al., 2014). This is demonstrated specifically by studies that show that current task-goals (Ansorge and Neumann, 2005; Kiefer and Martens, 2010; Schmidt and Schmidt, 2010), different forms of attentional selection (Naccache et al., 2002; Ansorge et al., 2009, 2010) and individual differences in attention and perception (Pohl et al., 2014; Prasad et al., 2017) modulate the extent of priming effects when the primes remain almost invisible. While the role of attention in masked priming has been studied using traditionally defined forms of exogenous and endogenous attention (Sumner et al., 2006; Ansorge et al., 2009, 2010), the role of newer forms of attentional control such as reward has not been examined.

In this study we are interested in investigating if barely perceived primes that are linked to rewards of different values modulate response selection when choices are made voluntarily. Masked primes have been shown to influence response selection during simple visuo-motor tasks (Neumann and Klotz, 1994; Leuthold and Kopp, 1998; Jaśkowski et al., 2002; Vorberg et al., 2003; Breitmeyer et al., 2004). Typically, a “prime” stimulus (eg., left or right arrow) is presented for a brief duration (typically <50 ms) followed/preceded by a mask to suppress the visibility of the prime. Participants are then presented with a target (eg., left or right arrow) stimulus to which they have to respond (eg., press left or right key). It is generally seen that responses are faster if the response required by the target (eg., left-arrow) matches the response required by the prime (eg., left arrow). In a free-choice variant of this paradigm pioneered by Schlaghecken and Eimer (2004), a neutral symbol not associated with any response (eg., a double-headed arrow) is presented as the target on some trials (followed by the primes) and the participants are asked to choose either of the responses (left or right key) on their own. In such a task scenario, participants tend to choose the response associated with the prime presented on that trial, even when they are supposed to be choosing “freely” (Kiesel et al., 2006; O'Connor and Neill, 2011; Ocampo, 2015; Prasad et al., 2017). These free-choice priming effects suggest that the primes trigger the associated response making the participants biased toward the primed-responses even when participants freely choose a response.

### Attentional Control Over Masked Visual Processing

Although there have been several demonstrations of masked priming of free-choice responses, it is not clear if this mechanism is completely beyond conscious control. Attentional involvement is usually not implicated since prime's influence has been taken to be “automatic” in the conventional sense. Posner and Snyder (1975) termed a process automatic if it proceeds in the absence of conscious awareness, is not susceptible to external influences and does not depend on capacity-limited resources. However, more refined theories of automaticity (Moors and De Houwer, 2006; Kiefer, 2007) have proposed the notion of conditional automaticity where unconscious processes are automatic to the extent that they proceed without any awareness and deliberation, but are initiated subject to the top-down goals of the individual and the availability of attentional resources. The direct parameter specification (DPS) proposed by Neumann (1990) is one such theory according to which unconscious stimuli can trigger an action plan only if it matches with the currently active task intentions. For instance, Ansorge and Neumann (2005) administered a task that involved responding to black or red targets. They observed priming effects for black primes only when participants searched for black targets (Experiment 1), but not when they searched for red targets (Experiment 2). Thus, only those features of the prime that were currently useful or relevant were processed suggesting that it is possible to strategically exert control over unconscious processing (see also, Schmidt and Schmidt, 2010; Tapia et al., 2013).

In line with this, attentional selection and prioritization as per current goals seem to modulate the prime's influence on response selection and action. Spatially or temporally attended primes lead to enhanced priming effects compared to unattended primes (Naccache et al., 2002; Sumner et al., 2006). For example, Naccache et al. (2002) observed that presenting targets on a predictable time scale (therefore facilitating temporal attention) lead to higher priming effects on a semantic categorization task. Taking into account this and similar findings (Jaśkowski et al., 2002; Kiefer and Brendel, 2006; Martens et al., 2011; see Ansorge et al., 2014 for a review), Kiefer et al. proposed the “attentional sensitization model of unconscious cognition” which suggests that processing of unconscious or briefly-visible information is susceptible to top-down control triggered by currently active task representations or attentional focus (Kiefer and Martens, 2010; Kiefer, 2012). This is achieved by enhanced sensitivity to processing in the task-relevant pathways and attenuation of the processing in task-irrelevant pathways. The model, thus, explicitly predicts that unconscious processes “should depend on available attentional resources” (p.1, Kiefer, 2012). But, “attention” encompasses many forms and can be deployed in many ways (Carrasco, 2011). It is not exactly clear which forms of attention are capable of modulating the extent of masked priming and which are not.

### Attentional Selection Mediated by Reward-Learning

Traditionally, attentional selection has been considered to depend on the physical properties of the stimulus (also referred to as stimulus-driven or exogenous form of attention, Theeuwes, 1992, 2010) and/or on the goals of the individual (Folk et al., 1992; goal-driven or endogenous form of attention, Egeth and Yantis, 1997; Theeuwes, 2010 for reviews; see Lamy et al., 2012). Apart from these, a third form of attentional control has been proposed recently (Anderson et al., 2011; Awh et al., 2012; Anderson, 2016a) which is mediated by selection history. Stimuli that have been previously attended-to receive priority in attentional selection. Thus, current goals, physical salience, and selection history all contribute to an integrated priority map (Theeuwes, 2019). Within this map, attentional priority is determined based on the strength of each of these individual factors which ultimately decides what is selected. Reward history is a form of selection history where attention is drawn to stimuli which were previously associated with reward. It is to be noted that the conceptualization of reward as a form of attention modulator is different from reward as a psychologically motivating factor. Motivational reward is directly linked to performance (eg., giving a chocolate to a child for completing homework) and is intended to incentivise certain type of behavior or responses. For instance, performance on a Stroop task (Padmala and Pessoa, 2010) or a Posner cueing task (Engelmann and Pessoa, 2014) is found to be enhanced when the participants are given reward contingent on their performance. Attentional reward, on the other hand, is inherently linked to certain aspects of a stimuli (feature or location) and does not depend on the participant's performance. Whether such reward-mediated attention is indeed a distinct form of attentional control (apart from endogenous and exogenous) or merely a variation of the existing categories has also been a matter of debate (Theeuwes, 2018, 2019). Nevertheless, it is now commonly understood that reward-associated stimuli can modulate attentional selection.

For instance, using a negative priming paradigm, Della Libera and Chelazzi (2006) showed that participants found it harder to respond to a stimuli whose inhibition was previously highly rewarded. Similarly, Munneke et al. (2015) showed that exogenous cues associated with higher reward lead to increased cueing effects on a Posner cueing task, compared to cues associated with lower reward. Such findings have suggested that reward-learning induces lingering biases associated with the rewarded stimuli which have been found to last days (Della Libera and Chelazzi, 2009), or even weeks (Anderson et al., 2011) after the initial association. In most such studies, the participants are made unaware of the reward contingencies either by having separate training and testing sessions or by inducing uncertainty in the reward associations. Also, the participants are typically (falsely) led to believe that the reward points depend on their performance. These measures are taken to ensure that the observed effects are due to biases in attentional selection and not due to strategic responses to the rewarded stimuli.

### Rationale for the Present Study

In this study, our interest was to examine whether free-choice priming is susceptible to influences by reward-learning. We adapted the masked priming design from Prasad et al. (2017) where the numbers “1” and “2” were presented as primes. The same numbers were presented as targets on forced-choice trials where participants pressed either “A” or “L” depending on the target. “0” was the target on free-choice trials where participants could choose between “A” or “L.” Free- and forced-choice trials were intermixed during presentation as it has been seen that free-choice priming effects are observed only when the response contingencies have already been established through forced-choice trials (Schlaghecken and Eimer, 2004). At the end of every forced-choice trial, reward points were displayed which was contingent on the target presented on that trial. That is, a reward of 10 points was given if the forced-choice target was “1” and 2 points were given for the target “2.” We also administered a control session with no reward points as a baseline measure of the masked priming effects.

On free-choice trials, participants were asked to freely choose between the response alternatives. Reward points were presented only on forced-choice trials, but not on free-choice trials. As a result, the forced-choice trials acted as the “training phase” which induced the reward-stimuli associations. The effect of these associations was dynamically tested on the free-choice trials. Several studies on reward-learning typically have a distinct training session in which the participants are trained with the reward association. The influence of this learning is subsequently examined in a testing phase without reward (see Anderson, 2016a for a review). We did not have two such separate sessions. Instead, reward learning induced in the forced-choice trials and its effects were tested dynamically on the free-choice trials since there is evidence to show that reward associations are learnt very quickly and their effects begin to show as early as on the subsequent trial (Hickey et al., 2010). Although, we present results from both free- and forced-choice trials, our primary interest was in examining if and how reward associations formed through forced-choice trials dynamically affect free-choice priming. We predicted that primes associated with higher reward would lead to enhanced free-choice priming effects on two key measures: proportion of choices and response times on free-choice trials. That is, we expected higher proportion of choices congruent with the prime on trials with high-reward primes. We also expected the RT (response time) priming effect (RT incongruent - RT congruent) to be greater for high-reward primes compared to the trials with low-reward and no-reward primes.

## Experiment 1

### Method

#### Participants

Twenty healthy participants (8 females, Mean age = 23.2 years, *SD* = 2.7) were recruited for the experiment. We conducted a power analysis to determine the required sample size by including studies that have shown masked free-choice priming effects (Schlaghecken and Eimer, 2004; Kiesel et al., 2006; Ocampo, 2015). Cohen's standardized difference scores (d_z_) were estimated using the reported paired-sample *t*-test values and sample sizes (i.e., d_z_ = t/√N; Cohen, 1988; Rosenthal, 1991; Hayward et al., 2018). The calculations were based on results reflecting differences between proportion of congruent and incongruent choices on free-choice priming trials since our main variable of interest was the choice proportion. Effect sizes estimated in this way ranged between 0.5 and 1. The power analysis for paired *t*-tests yielded sample sizes ranging from 10 to 33 for a desired power of 0.8 with the confidence level set to 0.05. We selected a sample size to fall within this range. Power analysis was performed using the “pwr” package in R.

All participants reported normal or corrected-to-normal vision and provided written informed consent. All the procedures of this experiment and the subsequent experiments were approved by the Institutional Ethics Committee (IEC) of University of Hyderabad.

#### Stimuli and Procedure

Stimuli were designed and presented using the SR research experiment builder (SR Research, Ontario, Canada) on an LCD monitor with resolution 1,024 * 768 pixels and refresh rate of 60 Hz. Participants were seated at a distance of 60 cm from the monitor. All stimuli were presented in black color (CIE-Lab: 0.00, 0.00, 0.00) against a gray background (CIE-Lab: 63.33, 0.00, −0.00). All the participants took part in two sessions: reward session and a control (no-reward) session. It has been seen that presenting no-reward trials has different consequences depending on whether they are embedded within a reward context or not (Munneke et al., 2015). In our study, the no-reward trials were administered as a separate control session to (1) demonstrate that masked free-choice priming effects on choices and RTs could be successfully obtained through our paradigm and (2) serve as a baseline for the reward session. The two sessions were administered on the same day with session order counterbalanced across participants.

##### Reward session

**Figure 1:**
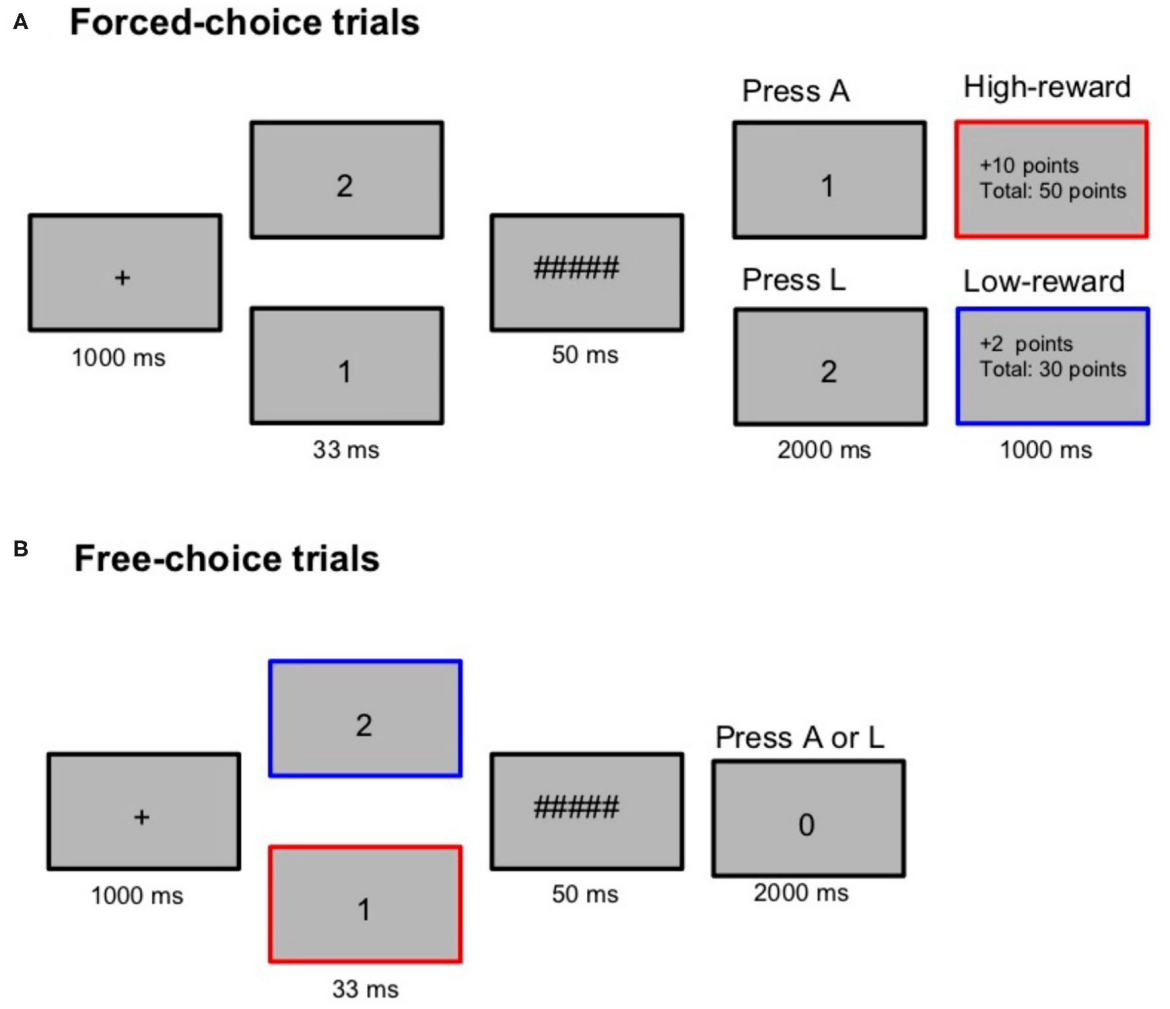
Trial structure during the reward session in Experiment 1. In the reward session forced-choice trials **(A)**, participants were required to press A on seeing the target “1” and press L on seeing the target “2.” Reward points (high: 10 or low: 2) contingent on the target were presented only on forced-choice trials. On free-choice trials **(B)**, they were asked to freely choose between A and L. The red and blue highlights were not present in the experiment. It is used here only to demonstrate that a high-reward target on a forced-choice trial (“1” in this example) is also a high-reward prime on a free-choice trial. A control session with similar trial structure (but without the reward points) was also administered with “3” and “4” as prime/target stimuli.

Throughout the trial, stimuli were presented in the center of the screen (Figure 1). The design was adapted from Prasad et al. (2017). Each trial in the reward session began with a fixation screen for 1,000 ms followed by a prime (the digit “**1**” or “**2**” in Times New Roman, pt. 26) presented for 33 ms. Following the prime presentation, a mask (“**######**,” Times New Roman, pt. 26) was presented for 50 ms. The target screen was displayed next for 2,000 ms or till a keypress was a registered. The target was either “**1**” or “**2**” on forced-choice trials and “**0**” on free-choice trials (Times New Roman, pt. 26). Participants were asked to press “A” on seeing “**1**” and to press “L” on seeing “**2**.” For the target “**0**,” they were instructed to freely choose and press either “A” or “L.” The target-response mapping was counterbalanced across participants. On free-choice trials, participants were instructed to choose spontaneously, “maintain some balance” while choosing and ensure that they don't choose one response most of the time. After the target disappeared, the reward earned on that trial and the total reward points were displayed on the screen, only on forced-choice trials. A reward of two points (low-reward) was given whenever the forced-choice target was “1.” Ten points (high-reward) were given when the forced-choice target was “2.” This mapping between the target and the reward-level was counterbalanced across participants. Five points were deducted on error trials. The participants were told that the reward points depended on their performance. To ensure that the participants don't explicitly become aware of the reward association, the reward contingency (between high/low reward and the targets 1/2) was reversed on 20% of the trials. A practice session of 40 trials was administered before the experiment.

The reward session consisted of two blocks of 200 trials each, for a total of 400 trials in the experiment. Each block consisted of 120 free-choice trials and 80 forced-choice trials with the masked primes divided equally between “1” and “2.” The free- and forced-choice trials were randomly mixed in each block. The target stimuli on forced-choice trials was also equally divided between “1” and “2” mapped to the two different reward-levels. Participants were given three self-paced breaks during the experiment.

##### Control session

The sequence of events in the control session were similar to those in the reward session except for the presentation of the reward points. The numbers “3” and “4” were used as prime/target stimuli. This was done to avoid possible carryover effects from the reward session (in those participants who performed the reward session first). The control session similarly consisted of two blocks with each block containing 200 trials (120 free-choice trials + 80 forced-choice trials). Twenty trials were given for practice before the main experiment.

##### Visibility tests

Two visibility tests of 120 trials each were administered after the reward and control sessions were both completed, to assess the visibility of the masked primes. The visibility tests were administered at the end to ensure that the participants don't explicitly become aware of the masked primes during the priming experiment. Henceforth, we will use “reward visibility test” to refer to the test with the primes “1” and “2”—used in the reward sessions. The “control visibility test” included primes “3” and “4.” The trial structure was similar to those in the priming experiments, except for the presentation of the target and the reward points. Instead of the target used in the priming experiments, participants were presented with “1” written in the center of a gray box (2°×2°) placed on the top-left corner of the display and “2” placed inside a box on the top-right corner of the display. Participants were instructed to click on the number that matched with the prime. Similarly, the numbers “3” and “4” were placed on the screen for the control visibility test. The primes were presented for 33 ms (masked trials) or for 200 ms (control trials). A different mode of response was selected for the visibility test to ensure that previously established stimulus-response mappings don't contribute to the responses leading to “priming of awareness” (Lin and Murray, 2014).

Each block of 120 trials consisted of 100 masked trials and 20 catch trials randomly intermixed. Catch trials with longer prime duration were included as a performance check to confirm if the participants understood the instructions and were attentive to the task (Lin and Murray, 2014). The mapping between the prime stimuli and its location on the display (left or right) was counterbalanced across participants. After each mouse click, the cursor was designed to re-position at the center of the screen. There was no time constraint for the responses. Twenty practice trials were given before the main experiment. Participants could also take a self-paced break halfway through the experiment.

### Results

Data from participants who chose one of the keys on more than 75% of the trials (four participants) was discarded as it violated our instruction to “maintain some balance” between both the key choices. Free- and forced-choice trials were analyzed separately. Trials with RT <150 ms and >2.5 median absolute deviation (MAD) away from the median RT (Leys et al., 2013) were excluded (Reward session: 8.1% of free-choice and 6.14% of forced-choice trials; Control session: 8.9% of free-choice and 7.5% of forced-choice trials). The MAD criterion was used because the common practice of discarding outliers 2–3 standard deviations away from the mean is unlikely to detect outliers correctly in small samples (Cousineau and Chartier, 2010). Trials with incorrect responses were also excluded (5.7 and 7% in the forced-choice trials of the reward and control session, respectively; there was no concept of “error” in free-choice trials). Mixed-effects models were used to analyse the data of all experiments using lme4 package in R environment (Bates et al., 2007). In all the analyses, random effects for Participants were included. The model outputs from all the analyses can be found in the Appendices A and B. Please see Table 1 for descriptive statistics.

**Table 1:** Descriptive statistics for free-choice trials (Experiment 1 and 2).

	**Experiment 1**			**Experiment 2**		
	**Choices**	**RT**		**Choices**	**RT**	
	**C**	**C**	**IC**	**C**	**C**	**IC**
High-reward	57.6 (4)	552 (4)	586 (5)	53.9 (5)	498 (4)	524 (4)
Low-reward	54.5 (6)	545 (3)	586 (5)	47.5 (5)	504 (4)	515 (4)
No-reward	59 (5)	538 (4)	617 (5)	50.9 (1)	536 (2)	549 (2)

*Choices are given in percentage and RT in ms. C, congruent, IC, incongruent. Only congruent choices are given because proportion of incongruent choices = 1 - C. The numbers in brackets denote 1 SE*.

#### Forced-Choice Trials

Forced-choice trials were analyzed first. A forced-choice trial was termed congruent if the prime matched the target. Congruency (congruent: −1, incongruent: +1) was added as a fixed effect to examine whether primes had any influence on the RTs. In the forced-choice trials, we were primarily interested in examining if the reward learning had taken place. To examine this, we analyzed the forced-choice RTs as a function of the “target-type.” One columns was created to compare high-reward with no-reward (HighNo: no-reward: −1, high-reward: +1) and another to compare low-reward to no-reward (LowNo: no-reward: −1, low-reward: +1). Target-type was determined based on the target-reward associations. For example, in one of the versions of the tasks, participants received high-reward when the target was 2 and low reward when the target was 1 in the forced-choice trials. Thus, for this version, all trials with target 2 were designated as target-type:high and trials with target 1 were designated as target-type: low. HighNo, LowNo and their interactions with congruency were entered as fixed effects in the mixed-effects analysis. The *p*-values of the effects were determined using Satterthwaite approximations to degrees of freedom, as implemented in the lmerTest function (Kuznetsova et al., 2017).

Accuracy analysis was performed on forced-choice trials by dummy coding responses into 1's (correct) and 0's (incorrect). Analysis was performed through generalized linear mixed-effects modeling (GLMM) using *glmer* function with family specified as binomial and link logit. Congruency and target-type were similarly sum-coded as in the RT analysis and entered as fixed effects. *p-*values were obtained through the default output of the glmer function in R based on asymptotic Wald tests (Luke, 2017).

#### Free Choices Trials

A response on a free-choice trials was termed “congruent” if the participant chose the key (eg., “A”) associated with the prime (eg., “1”) presented on that trial. If not (eg., “L”), the choice was termed “incongruent.” It is to be noted that “congruency” was only determined during the data analyses stage. Binomial tests comparing the proportion of congruent choices with chance (0.5) were first conducted using the binom.test function in R to examine if there was a global influence of the prime on free choices. Significantly higher proportion of choices indicate that the primes biased participants' responses irrespective of other factors. Next, the effect of other variables on congruency was examined using *glmer* function with family specified as binomial and link logit (similar to accuracy analysis). Congruency variable was dummy coded into 1's (congruent) and 0's (incongruent). Prime-type (HighNo and LowNo) factors were sum coded and included as fixed effects. The prime-type here indicates the reward associated with the numbers presented as primes. No target-related reward was given on free-choice trials. All main effects and interactions were included in the model. *p-*values were obtained through the default output of the glmer function in R based on asymptotic Wald tests (Luke, 2017). Mixed effects analyses were conducted on free-choice RT using the lmer function with congruency (congruent: −1, incongruent: +1), prime-type (HighNo, LowNo) and their interactions as fixed effects. The lmerTest analysis was similar to that of forced-choice RT. The effect of the order in which the reward session was administered was analyzed by entering order (reward session first: +1, reward session second: −1) as a fixed effect in the analyses^1^. The results of this analyses are reported in Appendix C.

Some studies have shown that effects of salience are short-lived (<300 ms) and disappear at longer response latencies (eg., Donk and van Zoest, 2008). Distributional analyses of the data was done using the vincentisation procedure (Ratcliff, 1979) to examine whether the effect of reward-learning was more evident during the faster responses. The free-choice RT data of each participant was arranged in ascending order and five bins were created by aggregating the data of each participant. Each bin comprised of the 25% of the data of each participant. Mixed effects analyses were conducted on choices and RT with Bin as the additional factor. Bin (Bin1, Bin2, Bin3, Bin4, Bin5) was dummy coded and entered as a fixed effect with Bin1 as the reference level. The interpretation of main effects in mixed effect model outputs is problematic when variables are dummy coded (Singmann and Kellen, 2019). For example, the main effect of congruency when Bin is entered as a dummy coded fixed effect refers to the difference between congruent and incongruent for Bin1 and not the difference between the congruency conditions averaged across ALL Bins which is we what we require. Thus, we will refer to the models without Bin for main effects of prime-type/congruency (in which all factors are sum-coded) and only look at the Bin related interactions in this analysis.

#### Prime-Visibility

Trials with responses faster than 150 ms and slower than 1,500 ms (Reward session: 8%, Control session: 5%) were discarded from the analysis. Accuracy on the catch trials was calculated first to assess if the participants understood the instructions and attended to the task. The prime “1” was arbitrarily designated as the signal and “2” was designated as the noise. Thus, correct responses to the prime “1” were considered as Hits and incorrect responses to the prime“2” were considered as False Alarms (FAs). Hits and FAs were corrected using the log-linear rule to adjust for occurrences of Hits and FAs of 0 or 1 (Hautus, 1995). Hit rate was calculated by dividing the number of Hits by the total number of signal trials. Similarly, FA rate was the number of FAs divided by the total number of noise trials. We computed d' on the masked trials for each participant as the difference between the z transform of Hit rate and the FA rate using the qnorm function in R. *t*-tests comparing the d' values with chance performance (d' = 0) were conducted to assess the visibility of the primes. Paired *t*-tests were also conducted to examine the difference in prime visibility between the reward and control sessions.

### Results

#### Forced-Choice Trials

**Figure 2:**
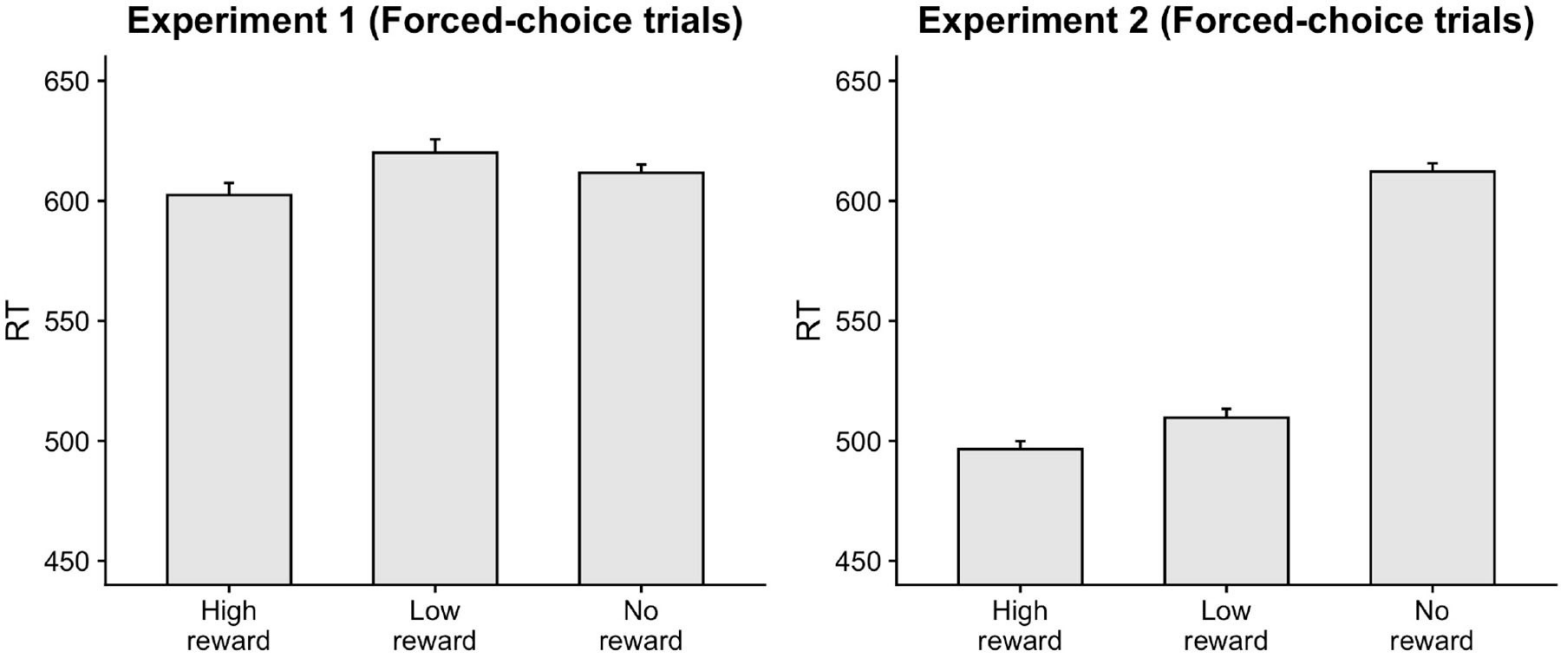
Faster responses were seen on forced-choice trials for targets associated with high-reward compared to no-reward.

The responses on congruent trials were faster compared to incongruent trials, β = 19.79, *t* = 8.83, *p* < 0.001. Accuracy was also greater on congruent trials, β = −0.43, *z* = −6.07, *p* < 0.001. These results show a clear facilitatory influence of the masked primes. Importantly, participants were faster and more accurate responding on trials where the target resulted in high-reward as opposed to no-reward, as indicated by a main effect of target-type (RT: β = −7.73, *t* = −2.35, *p* = 0.019; Accuracy: β = 0.27, *z* = 2.43, *p* = 0.015) confirming that participants indeed associated the numbers with the given reward points in the forced-choice trials (Figure 2). Interestingly, responses on low-reward trials were slower than no-reward trials, β = 8.53, *t* = 2.57, *p* = 0.01. Below, we examine the effect of reward-learning on the priming effect in free-choice trials.

#### Free-Choice Trials

**Figure 3:**
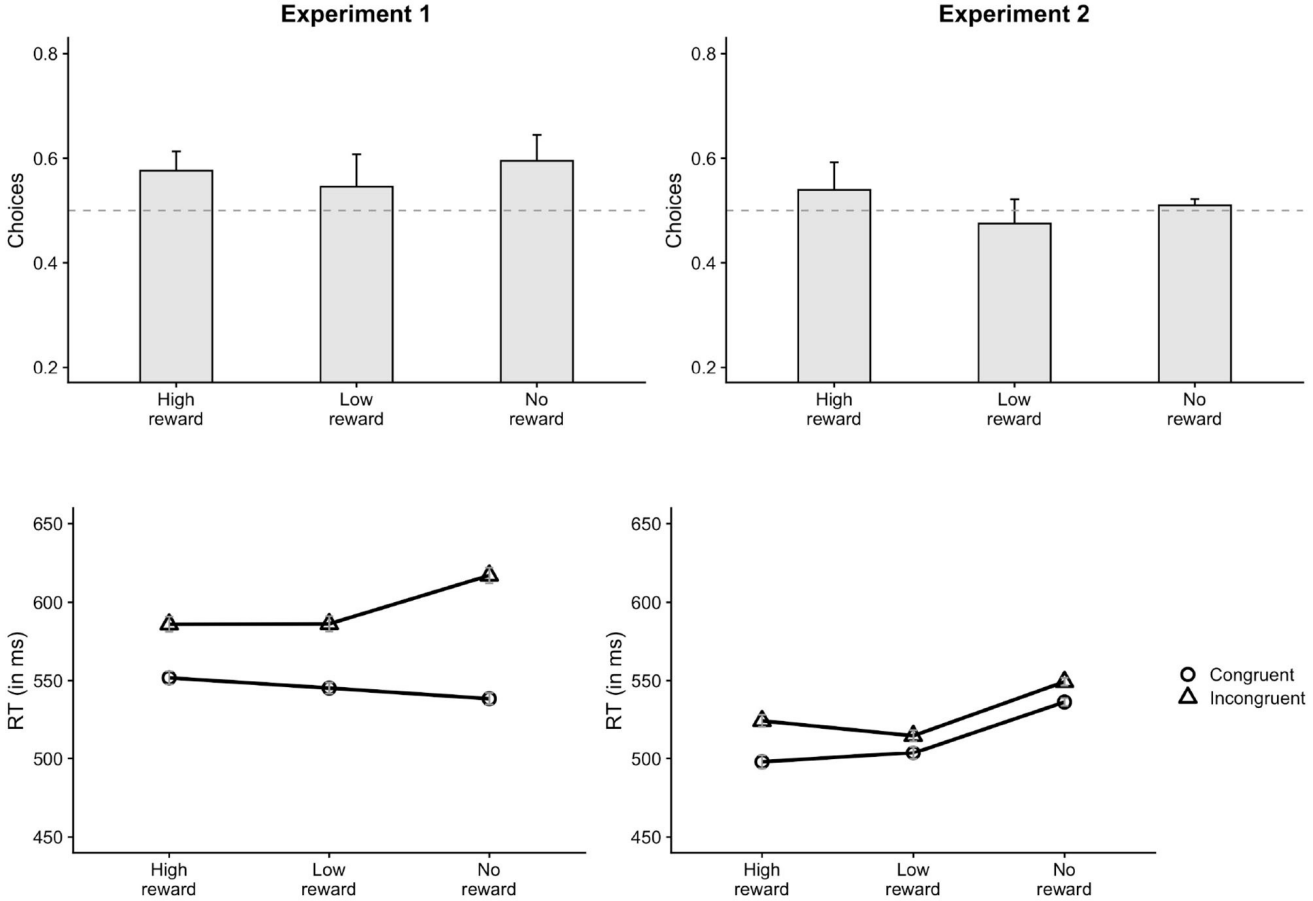
Results of Experiment 1 and 2. In Experiment 1 (left), participants chose the congruent responses less often on low-reward trials compared to no-reward trials. The RT priming effect on low-reward trials was also lesser than no-reward trials. In Experiment 2 (right), congruent choices on high-reward trials were greater than no-reward trials. Congruent choices on low-reward trials were lesser compared to no-reward trials. No effect of reward was observed on RT priming effect. “No-reward” corresponds to the control session trials. Error bars indicate ± 1 SE. The horizontal dashed line in the choice plot indicates chance level (50%).

**Figure 4:**
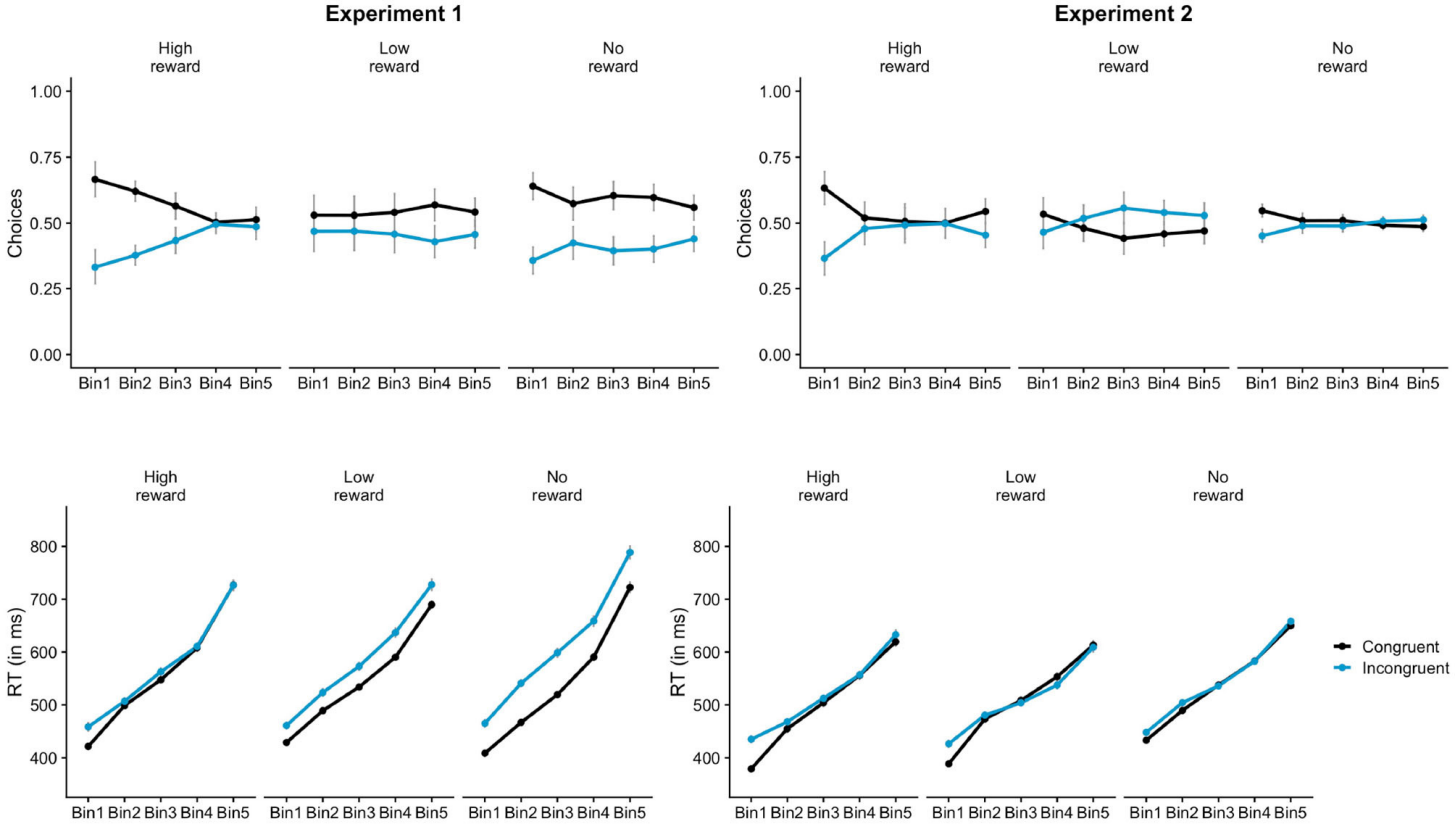
Distributional analysis of free-choice trials in Experiment 1 and 2. In Experiment 1 (left), participants chose the congruent response more often when the primes were associated with high-reward compared to no-reward only in the faster responses (Bin1 and Bin2). RT priming effects on reward trials (high and low both) were consistently lesser compared to no-reward across all Bins. In Experiment 2 (right), there were more congruent choices on high-reward trials compared to no-reward during the fastest responses (Bin1). The effect was diminished in the subsequent bins. RT priming effect was greater for high-reward primes compared to no-reward in the fastest responses (Bin1). Similar effect was seen for low-reward primes in Bin2.

Participants chose the congruent response (59.7%) more often than the incongruent response in the control session (*p* < 0.001). The proportion of congruent responses (56.1%) were also significantly greater than chance in the reward session (*p* < 0.001). This indicates that the primes had a strong influence on the choice behavior in both reward and control sessions. The lmer analysis on the choice data further showed that the proportion of congruent choices for low-reward primes was lesser than no-reward primes, as shown by a significant main effect of prime-type for LowNo condition, β = −0.12, *z* = −2.91, *p* = 0.004 (Figure 3). There was no main effect of HighNo condition, β = 0.01, *z* = 0.33, *p* = 0.744. The distributional analysis with five Bins showed a significant interaction between Bins and reward conditions for both HighNo (Bin3: β = −0.31, *z* = −2.41, *p* = 0.016, Bin4: β = −0.59, *z* = −4.54, *p* < 0.001, Bin5: β = −0.44, *z* = −3.43, *p* < 0.001) and LowNo conditions (Bin3: β = 0.24, *z* = 1.92, *p* = 0.054, Bin4: β = 0.43, *z* = 3.36, *p* < 0.001, Bin5: β = 0.41, *z* = 3.19, *p* = 0.001). In Bin1 and Bin2, there were more congruent choices associated with high reward primes (Bin1: β = 0.06, *z* = 3.44, *p* < 0.001, Bin2: β = 0.04, *z* = 2.18, *p* = 0.029) and fewer choices associated with low-reward primes compared to no-reward primes (Bin1: β = −0.08, *z* = −4.18, *p* < 0.001, Bin2: β = −0.04, *z* = −2.2, *p* = 0.028, Figure 4). There was no effect of reward condition in Bin3 (*p* > 0.2). In Bin4, high-reward primes lead to fewer congruent choices compared to no-reward (β = −0.06, *z* = −3.07, *p* = 0.002). There was no effect of low-reward in Bin4 (*p* > 0.5). Neither of the reward conditions had any effect in Bin5 (*p* > 0.1).

Analysis of free-choice RTs showed faster RT for congruent incongruent responses compared to incongruent responses, β = 5.79, *t* = 3.46, *p* < 0.001. There was a significant interaction between prime-type and congruency (LowNo: β = −11.06, *t* = −4.66, *p* < 0.001) revealing substantially lower priming effects for low-reward primes compared to no-reward primes (Figure 3). No such interaction was observed for the HighNo condition (β = −0.07, *t* = −0.03, *p* = 0.976). There was a main effect of prime type (LowNo: β = −4.6, *t* = −1.96, *p* = 0.05) indicating that responses on trials with low reward-associated primes were faster compared to the control condition. The distributional analysis show a significant three-way interaction between HighNo, Bin and congruency (Bin5: β = −17.84, *t* = −4.13, *p* < 0.001). The interaction between LowNo condition, congruency and Bins were also significant (Bin5: β = 10.43, *t* = 2.45, *p* = 0.014). Separate models were created for each Bin. The interaction between congruency and LowNo condition was significant in all the Bins except Bin5 (Bin1: β = −7.47, *t* = −3.41, *p* < 0.001, Bin2: β = −7.70, *t* = −4.28, *p* < 0.001, Bin3: β = −8.51, *t* = −4.24, *p* < 0.001, Bin4: β = −9.46, *t* = −3.47, *p* < 0.001) indicating reduced priming effects for low-reward trials compared to no-reward trials (Figure 4). Similar reduction in priming effects for high-reward trials compared to low-reward trials was seen for Bin2, Bin3, and Bin5 (Bin2: β = −3.91, *t* = −2.13 *p* = 0.034, Bin3: β = −5.41, *t* = −2.62, *p* = 0.009, Bin5: β = −12.97, *t* = −3.01, *p* = 0.003).

### Visibility Test (Reward and Control Sessions)

Participants performed very well on the catch trials in the reward and the no-reward sessions (Reward: *M* = 95.2%, *SD* = 5.3; Control: *M* = 97.4 %, *SD* = 3.6). The prime visibility index d' deviated significantly from zero for both reward and control sessions (Reward: *d'* = 1.37, *SD* = 0.59, *t*_(1, 15)_ = 9.4, *p* < 0.001, Control: *d'* = 1.52, *SD* = 0.5, *t*_(1, 15)_ = 12.18, *p* < 0.001). Prime visibility did not differ between Reward and control (without reward) sessions, *t*_(1, 15)_ = −0.93, *p* = 0.368.

### Discussion

The control experiment replicated free-choice and forced-choice priming effects observed in several previous studies (eg., Kiesel et al., 2006; Prasad et al., 2017). The masked primes lead to higher proportion of congruent choices (on free-choice trials) and faster response times on congruent trials (on free- and forced-choice trials). This confirms that our design was capable of capturing masked priming effects. The analysis of the forced-choice trials of the reward session showed faster responses for visible targets associated with high-reward compared to no-reward confirming that participants learnt to associate reward with the numbers presented on the forced-choice trials.

The free-choice trials of the reward session were then compared with those of the control session to examine the effects of reward-learning on free-choice priming effect. The analysis on the proportion of choices showed that participants chose the response associated with high-reward primes more often than no-reward primes, but only in the faster responses (Bin1 and Bin2). In the slower responses (Bin4), the opposite pattern was observed. The effect of the prime on the choices was also lower for the low-reward condition compared to no-reward in the faster responses (Bin1 and Bin2). The RT data also showed a similar pattern. Priming effects (RT on incongruent trials—RT on congruent trials) on both high-reward and low-reward trials were substantially lower than priming effects on no-reward trials, irrespective of whether the responses were faster or slower.

These findings provide evidence for reward-mediated control on masked priming effects. The choice data indicates that reward-learning facilitated priming effects for the faster responses, but as the responses became slower the effect of reward-learning diminished. Faster RTs are generally considered to capture the saliency effects while longer RTs are considered to be a result of top-down processes (Theeuwes, 2018). Thus, this result is in line with studies that have shown that reward primarily modulates the perceptual saliency of the associated objects (Hickey et al., 2010).

## Experiment 2

The primary objective of Experiment 2 was to replicate the findings of Experiment 1 with a different set of prime and target stimuli. This was deemed necessary because the numbers used for reward levels (“2” and “10”) were similar to the prime/target stimuli (“1” and “2”). It is possible that this similarity lead to or at least confounded the observed effects in Experiment 1. Thus, in Experiment 2, we tested the effect of reward levels using arrows as prime/target stimuli. Arrows are the most commonly used stimuli in masked priming paradigms (eg., Eimer and Schlaghecken, 2003; Kiesel et al., 2006; Atas and Cleeremans, 2015). If reward value indeed modulates masked-priming effects irrespective of the type of stimuli used, we expected to replicate the findings from Experiment 1.

### Method

#### Participants

Twenty healthy participants (10 females, Mean age = 24 years, *SD* = 2.6) took part in Experiment 2. None of them had participated in Experiment 1.

#### Stimuli and Procedure

The trial structure and timing in Experiment 2 was exactly the same as in Experiment 1. The only difference was that the prime and target stimuli in the reward sessions were replaced by arrows. Participants could be presented with “**<**” or “**>**” as primes. The same arrows were also presented as targets on forced-choice trials. “** <>**” was presented as the target on free-choice trials. The mask was same as that used in Experiment 1. Participants were asked to press “A” on seeing “**<**” and “L” on seeing “**>**.” This mapping was not counterbalanced to avoid potential slowing down due to the spatial incongruency between the arrow direction and response key location in the counterbalanced version. The mapping between reward value and target was counterbalanced as in Experiment 1. In the control session, up (“**∧**”) and down (“_**V**_”) arrows were used as prime/target stimuli on forced-choice trials and their combination was presented as the target on free-choice trials. “A” and “L” were used as response keys and the target—response mapping was counterbalanced across participants. The order of reward and control sessions was also counterbalanced across participants. In the end, participants took part in visibility tests corresponding to the reward and control sessions.

The number of trials in each session and the blocking levels were same as in Experiment 1.

### Analyses

Three participants' data was excluded because they chose one of the responses more than 75% of the times in free-choice trials. One participant did not complete the control session. Thus, the final analyses included data from 16 participants. The data analyses procedure for all the variables was similar to that used in Experiment 1 (see Appendix B for the model outputs and Table 1 for descriptive statistics). Outliers were discarded in the reward session (free-choice: 9%; forced-choice: 5%) and the control session (free-choice: 9.1%; forced-choice: 6.4%) based on the MAD criterion. Incorrect trials were also excluded from the analysis (reward: 3%; control: 4.6%). In the prime visibility test, 4.5% of trials in the reward session and 7% of trials in the control session were discarded as outliers.

### Results

#### Forced-Choice Trials

Congruent responses were executed faster and more accurately than incongruent responses, (RT: β = 8.48, *t* = 3.46, *p* < 0.001; Accuracy: β = −0.26, *z* = 2.73, *p* = 0.006. Participants were also faster responding on trials with high-reward and low-reward target-type compared to no-reward trials (HighNo: β = −45.78, *t* = −12.62, *p* < 0.001; LowNo: β = −32.88, *t* = −9.03, *p* < 0.001). The accuracy on high-reward trials was also greater than no-reward trials, β = 0.46, *z* = 3.07, *p* = 0.002. These results confirm that participants indeed associated the target stimuli with the given reward points (Figure 2).

#### Free-Choice Trials

Binomial tests showed that the proportion of congruent choices was not significantly different from chance (0.5) for both reward (50.7%), *p* = 0.417 and control sessions (50.9%), *p* = 0.249. The mixed-effects analysis on the proportion of congruent choices revealed a significant effect of prime-type. These main effects show that participants chose the congruent response more often for the high-reward primes compared to the no-reward primes (HighNo: β = 0.12, *z* = 3.23, *p* = 0.001, Figure 3). But, the proportion of congruent choices were lower for low-reward primes compared to no-reward primes (LowNo: β = −0.13, *z* = 3.46, *p* < 0.001). The distributional analysis revealed a marginally significant interaction between HighNo and Bin2 (β = −0.22, *z* = −1.8, *p* = 0.072). Separate models showed greater congruent choices for high-reward trials compared to no-reward trials in Bin1 (β = 0.06, *z* = 2.9, *p* = 0.004) and Bin5 (β = 0.04, *z* = 2.07, *p* = 0.038, Figure 4). The opposite effect was observed for LowNo condition, only in Bin3 (β = −0.05, *z* = −2.3, *p* = 0.021).

The free-choice RTs were faster on congruent trials compared to the incongruent trials indicating a global influence of the masked prime on response-times (β = 4.31, *t* = 4.01, *p* < 0.001). There was a significant main effect of prime-type (HighNo: β = −9.85, *t* = −6.2, *p* < 0.001; LowNo: β = −11.48, *t* = −7.23, *p* < 0.001) indicating that responses on trials with reward-associated primes were faster than the control trials. The interactions between congruency and prime-type (Figure 3) was neither significant for the HighNo condition (β = 2.15, *t* = 1.31, *p* = 0.189) nor for the LowNo condition (β = 0.05, *t* = 0.03, *p* = 0.974). The distributional analysis showed a significant three-way interaction between prime-type, congruency and HighNo condition (Bin2: β = −5.33, *t* = −2.08, *p* = 0.037, Bin3: β = −5.27, *t* = −2.07, *p* = 0.038, Bin4: β = −7.49, *t* = −2.9, *p* = 0.004; Bin5: β = 6.48, *t* = −2.54, *p* = 0.011). The three-way interaction between LowNo, congruency and Bin was marginally significant (Bin5: β = 64.65, *t* = 1.84, *p* = 0.065). Separate models created for each Bin showed marginally greater priming effects for high-reward trials compared to no-reward trials only in Bin1 (β = 3.06, *t* = 1.66, *p* = 0.097, Figure 4), but not in the subsequent Bins (*p* > 0.4). Greater priming effects for low-reward trials compared to no-reward were also seen in Bin2 (β = 2.06, *t* = 1.71, *p* = 0.088).

### Visibility Test (Reward and Control Sessions)

The mean accuracy on the catch trials in the reward and the control session was 97% (*SD* = 5.7) and 96 % (*SD* = 7.2), respectively. The prime visibility index deviated significantly from zero in the reward (Mean *d'* = 1.69, *SD* = 0.69, *t*_(1, 15)_ = 9.72, *p* < 0.001) as well as the control sessions (Mean *d'* = 1.62, *SD* = 0.73, *t*_(1, 14)_ = 8.9, *p* < 0.001). The paired *t*-test showed no significant difference between the discrimination performance in the reward and control sessions, *t*_(1, 15)_ = −0.38, *p* = 0.711.

### Discussion

Experiment 2 replicated the main finding of Experiment 1—primes associated with higher reward lead to higher free-choice priming effects compared to no-reward primes in the faster responses. This effect was seen both in proportion of choices and RT data. Participants chose the response associated with the high-reward prime more often compared to no-reward prime in faster responses. Similarly, priming effects was greater for both high-reward primes and low-reward primes compared to no-reward primes in the faster responses. In slow responses, priming effect for high- and low-reward primes was equivalent to that of no-reward primes. The results of both experiments together provide converging evidence for the role of reward value on masked free-choice priming.

## General Discussion

In two experiments, we examined whether high- and low- reward values associated with masked primes modulated free-choice priming effects. The reward-learning was induced by target-contingent reward on forced-choice trials. We tested with both number (Experiment 1) and arrow primes (Experiment 2). In both experiments, we observed higher proportion of congruent choices for primes associated with high-reward as opposed to no-reward. This was observed predominantly in the faster responses in both experiments. The RT priming effect in free-choice trials was consiststently reduced for high-reward condition compared to no-reward in Experiment 1. In Experiment 2, priming effect for both high- and low-reward primes were greater than no-reward primes, but this was only observed for the faster responses.

To our knowledge, this is the first set of findings that show the susceptibility of masked priming effects to reward-mediated attentional control. Most studies so far have shown that the extent of the influence of masked primes depends on current task expectations, spatial and temporal attention, and other factors. We propose an additional factor, in the form of reward-learning, capable of modulating masked visuomotor priming. These findings are also in line with recent studies that have shown that masked visual processing is flexible and susceptible to top-down control (Kiefer, 2012). Models such as the attentional sensitization model have proposed that while certain unconscious processes proceed in a manner that can be considered automatic, they are triggered subject to currently active task representations, deployment of attentional resources, and other factors (Ansorge et al., 2014). In line with this, we find that reward-mediated attentional selection can modulate masked priming of free-choices.

The influence of reward-learning on free-choice priming was evident in the faster responses (in the choice data in Experiment 1 and 2, and RT data in Experiment 2). Both neurophysiological and behavioral evidence exists to suggest that reward drives attentional selection by modulating saliency of the perceptual representations of reward-associated stimuli (Hickey et al., 2010; Bucker et al., 2015; Failing and Theeuwes, 2018). Such saliency-based effects are expected to be short-lived. In fact, Donk and van Zoest (2008) showed that saliency-driven attentional selection is prominent up until ~350 ms after the onset of the stimulus. Thus, our results are in line with this proposal and suggest that reward modulates perceptual salience of the masked primes and biases selection by favoring the competition toward rewarded masked primes in the early stages of visual processing. However, it is to be noted that our tentative conclusions are based on behavioral data. Neurophysiological evidence would be necessary to determine exactly which stage of visual processing during masked priming is modulated by reward-learning.

The finding of increased reward-mediated effects in the faster responses is also consistent with rapid-chase theory (Schmidt et al., 2006) and the feedforward model of masked priming (Lamme and Roelfsema, 2000). According to these, priming effects are a result of the feedforward signals from the prime and the target which proceed without the involvement of conscious report. The prime independently drives responses initially which are then taken over by the target. As responses get slower, an inhibitory mechanism is triggered against the primed responses leading to reduced or inverse priming effects. Our results are in line with these predictions (Sumner, 2007; Panis and Schmidt, 2016). In both the studies, RT and choice priming effects were reduced or non-existent in Bins4 and 5.

The results from RT data in Experiment 1 seem to be inconsistent with this explanation. RT priming effects were reduced for high-reward primes (compared to no-reward) even for the fastest responses which is in contradiction with the saliency-based explanation. We would have expected higher RT priming effects for high-reward condition in the faster responses. This is possibly related to the finding that the reward-related effects were stronger in Experiment 2 compared to Experiment 1. This is supported by considerably faster RT for both high- and low-reward targets compared to no-reward (high: 496 ms, low: 510 ms, no: 612 ms) in the forced-choice trials in Experiment 2. In contrast, the difference between high-reward (602 ms) and no-reward (612 ms) targets on forced-choice trials was much lesser in Experiment 1 suggesting that reward-learning on forced-choice trials was stronger in Experiment 2 compared to Experiment 1. Further, reward-related effects persisted in the free-choice data even for slow responses in Experiment 2, whereas they disappeared for the slow responses in Experiment 1. Thus, since the efficiency of reward-learning on forced-choice trials in Experiment 1 was weak to begin with, it is possible that its subsequent effects on free-choice trials dissipated quickly resulting in reduced effects on response times. It is also possible that the participants inhibited the prime-related response to follow the instruction to maintain “some balance” while choosing. This could also explain the inconsistent effects across different time bins in both the experiments. Another potential reason for the inconsistent effects could be low power of our experiments. Although we performed a power analysis, we selected a sample size (20) well within the upper limit of the desired range (33). Further, apart from the number of participants, the total number of trials per each participant also contributes to the overall power of a study. We acknowledge that we might have observed more robust effects with an increased number of participants/trials.

In the choice and RT analysis of both the experiments, barring few exceptions, the effect of reward-learning was generally lower for low-reward condition compared to no-reward. This could be because we administered reward and no-reward trials (control session) in separate blocks. Thus, in the reward session, participants either got high- or low-reward which could have lead to the devaluation of the low-reward trials. We might have observed a graded effect (high > low > no) if we had intermixed no-reward trials with reward trials (Munneke et al., 2015 for one such example).

It is important to note that interpreting the role of reward-learning on forced-choice trials in our study is not straight-forward. On every forced-choice trial, the prime could be linked to either high- or low-reward. Similarly, the target could also be associated with either high- or low- reward. Thus, high-reward targets are greatly facilitated by congruent primes (which are also high-rewarded) as opposed to low-reward targets which are poorly facilitated by congruent primes (which are low-rewarded). This is an artifact that complicates interpreting the effect of reward-learning on forced-choice priming. The origin of the problem is obvious. We used the forced-choice trials to train the reward associations in the participants. One way to get around this problem is to have a separate training session where the prime/target stimuli are associated with reward points. While this doesn't completely solve the problem, the issues caused by immediate appearance of reward points following forced-choice targets can be avoided. Another, probably more efficient way would be to associate reward with prime locations so that the strength of prime processing is modulated independent of the target (Sumner et al., 2006; Schmidt and Seydell, 2008).

It is possible to question whether the reward was getting associated with the prime or the target since both were displayed on every forced-choice trial before the reward was presented. We believe that the reward was associated with the targets since the number of points gained depended on the type of target. There was no pattern associated with the presentation of the primes. Hence, it is not possible for any systematic learning to have taken place associating the reward levels with the type of prime. Another potential concern could be that we did not have a separate training sessions to induce reward associations. One of the criticisms against evaluating such dynamic learning of reward values through a single experiment where the rewarded stimuli is also task-relevant (instead of having distinct training and testing sessions) is that participants might strategically select reward-predictive stimuli. Thus, these effects might indicate motivation-driven response behavior rather than a reward-mediated alteration in the attentional bias. However, this criticism may not apply to our results because reward points were not presented on the free-choice trials. Further, the reward-predictive stimuli (that is, “1” and “2”) were not task-relevant on the free-choice trials as the participants only had to respond to the free-choice target (“0”) and the reward-associated stimuli (“1” and “2”) were masked.

Still, it is possible to question if the observed effects were indeed because the rewarded-primes triggered the corresponding responses more often. Instead, it can be said that participants simply chose the key-press (on a free-choice trial) that previously resulted in a high-reward (based on their experience with forced-choice trials), irrespective of the prime's influence. Thus, it is possible that the observed effects were not mediated through the primes and instead, reward directly influenced the responses. For instance, participants could have pressed A more often on free-choice trials (irrespective of the prime) simply because they received high-reward whenever they pressed A (on forced-choice trials). We do not think is likely as the reward contingency on the forced-choice trials was reversed on 20% trials to prevent any such explicit strategies from being formed. We also analyzed our data to rule out this confounding explanation of our effects. We tested if the proportion of trials on which the participants chose the response associated with reward (irrespective of the prime) was significantly greater than chance (See Appendix D for detailed description of the analyses). In both the experiments, participants did not choose the response associated with reward significantly more than chance, (Experiment 1: *t*_(1, 15)_ = 0.41, *p* = 0.69; Experiment 2: *t*_(1, 15)_ = 1.5, *p* = 0.157). Thus, we can tentatively rule out prime-independent explanations for the reward-based effects seen in our study. However, since this is one of the first studies to examine the role of reward-learning on free-choice priming, we acknowledge that further studies are necessary to draw strong conclusions and rule out alternate explanations.

The objective of this study was to investigate the influence of reward-learning on masked free-choice priming. Are such free-choice priming effects restricted to response priming paradigms with identical primes/targets as used in this study? Testing the influence of the prime independent of the response can be done, for example, by using the semantic priming paradigm. Interestingly, free-choice priming effects have been demonstrated in semantic priming as well. In one such study, Ocampo (2015) administered a free-choice version of the classic number magnitude judgement task by Dehaene et al. (1998). On forced-choice trials in this task, participants saw a target (2, 4, 7, or 9) and were asked to press the left button if the target was <5 and press the right button if the target was >5. On free-choice trials, people could freely choose between the two responses. Either novel (eg., 3 or 8) or repeat (eg., 2 or 7) primes were presented before the target. As expected, congruency effects were seen on forced-choice trials. Importantly, on free-choice trials, participants chose the prime-congruent responses more often and were faster while doing so. This effect was the same for novel and repeat primes suggesting that existing stimulus-response links do not necessarily enhance free-choice priming effects.

A possible limitation could be our use of points as reward. Most studies on reward-mediated attention have used financial reward where participants are given money based on their final reward scores (eg., Della Libera and Chelazzi, 2006; Anderson et al., 2011; Munneke et al., 2015). Here, we used just the display of reward points to manipulate attention. Although we did find the expected effects even without the use of financial reward, it is possible to question whether the mere display of points-earned is enough to manipulate attentional selection. Several studies have shown that reward does not always have to be monetary and that other tokens such as points (Shomstein and Johnson, 2013), food (Pool et al., 2014), and social reward (Anderson, 2016b) can have a similar influence. The mere presentation of numerical reward points as feedback has the same influence as providing monetary reward on complex behavior such as anticipatory control (Adam et al., 2012). These findings can be justified based on the idea of a common neural currency in the brain (Levy and Glimcher, 2012) according to which we encode the subjective value of different type of rewards on a common scale.

The discrimination performance on the visibility test was significantly greater than chance level performance in Experiment 1 and 2. Thus, the effects observed here can't be confidently generalized to stimuli that are completely below the threshold of awareness. Several masked priming studies with brief stimuli aiming to examine unconscious processing have similarly observed above-chance performance in the prime visibility test (eg., Sumner, 2008; Pohl et al., 2014; Prasad et al., 2017). It is to be noted that the objective prime visibility test is an overestimation of the true visibility of the primes during the experiment. This is because in the visibility test, the participants are explicitly informed about the primes making them task-relevant which could create an attentional template to look for the prime. These conditions are different from those in the main experiment where the participants are completely unaware of the nature of the primes. Further, a dissociation between the extent of prime awareness and the priming effect is commonly observed (eg., Mattler, 2003; Francken et al., 2011) suggesting that priming effects can be independent of the visibility of the primes.

More recently, Koivisto and Neuvonen (2020) observed above-chance performance on an objective prime discrimination task even when participants subjectively reported to have seen “nothing.” Further, it has been shown that high prime-target similarity can lead to lower discrimination performance on the visibility test. This could be due to confusions and mistakenly responding to targets instead of primes on incongruent trials (Khalid et al., 2011). To avoid this, we did not present the target in the visibility tests. But this gives rise to another problem: it violates the exhaustiveness criteria for unconscious processing (Reingold and Merikle, 1988). That is, the prime visibility test was not exactly same as the main priming task. Several researchers have suggested that the prime visibility test must be identical with the main priming task because differing task demands can modulate the visibility measure (Eriksen, 1960). This could be a serious limitation of our study. All these points indicate that it is necessary to conduct more studies with stricter control on the prime awareness measure. The diversity of methods in the existing literature to induce lack of awareness and measure it has lead to controversies on the most suitable method (Rothkirch and Hesselmann, 2017). One possible alternative, other than using objective visibility tests like the one used in this study, is to rely on subjective measures of awareness such as the perceptual awareness scale (PAS, Ramsøy and Overgaard, 2004). In sum, we acknowledge that our awareness measures could have limitations which prevent us from making strong conclusions about reward-related influences on “unconscious” processing.

## Conclusion

The relationship between attention and awareness is one of the mostly intensely debated topics in cognitive science. Thus, determining the sources of attentional capture and selection has implications not just for research on masked processing, but to general theories of attentional selection as well. In this set of experiments, we show the influence of reward-mediated attentional selection on masked priming of free-choices. Although these results are preliminary and need further replications, they add to the growing literature concerning the depths and limits of masked visual processing.

## Data Availability Statement

The datasets generated during and analyzed during the current study are available in the OSF repository: https://osf.io/utpzx/?view_only=bc0890abfc1c4550a3750fc3f1f1f948.

## Ethics Statement

The studies involving human participants were reviewed and approved by Institutional Ethics Committee (IEC) of University of Hyderabad. The patients/participants provided their written informed consent to participate in this study.

## Author Contributions

SP and RM conceived the experiment and edited and revised the paper. SP collected the data, performed the analysis, and wrote the paper. Both authors contributed to the article and approved the submitted version.

## Conflict of Interest

The authors declare that the research was conducted in the absence of any commercial or financial relationships that could be construed as a potential conflict of interest.

## Footnotes

^1^We thank an anonymous reviewer for this suggestion.
