# Supplementary material for: Reward Influences Masked Free-Choice Priming
Source: Front Psychol. 2020 Nov 30;11:576430. doi: 10.3389/fpsyg.2020.576430 (PMC7733960; doi:10.3389/fpsyg.2020.576430)
Supplement: Supplementary file 1 [file Data_Sheet_1.pdf]

Prasad, S. G., & Mishra, R. K. (2020). Reward influences masked free-choice priming.
Frontiers in Psychology

Appendix

Results from mixed effects analysis in Experiment 1 - 2. In all the analyses, fixed effects were analysed using sum-coding, except for the “Bin. In the analysis of choice and accuracy data, the dependent measures were dummy-coded into 1’s and 0’s (Congruent: 1, Incongruent:0; Correct: 1, Incorrect: 0). Random effects were included for Participants. For more details on the analysis, please see the “analysis" section of Experiment 1 in the main text. Below, we mention the fixed-effects variables included in the analysis and the levels of coding.

**Variables**	**Levels & Coding**
target_HighNo	high: +1, no: -1
target_HighNo	low: +1, no: -1
Bin	Bin1 (reference), Bin2, Bin3, Bin4, Bin5
prime_HighNo	high: +1, no: -1
prime_LowNo	low: +1, no: -1
congruency	congruent: -1, incongruent: +1

A. Experiment 1.

A1. Forced-choice trials

	Dependent variable:	
	RT (1)	Accuracy (2)
congruency	19.789	-0.435
	t = 8.834***	t = -6.068***
target_HighNo	-7.727	0.267
	t = -2.336*	t = 2.428*
target_LowNo	8.530	-0.147
	t = 2.568*	t = -1.467
congruency:target_HighNo	1.386	0.067
	t = 0.419	t = 0.612
congruency:target_LowNo	-5.942	0.100

	t = -1.789	t = 1.003
Constant	613.784	3.331
	t = 25.511***	t = 10.973***
Observations	4,470	4,772
Log Likelihood	-28,531.810	-985.424
Akaike Inf. Crit.	57,081.610	1,986.848
Bayesian Inf. Crit.	57,139.260	2,038.612
Note:	#p<0.1, *p<0.05; **p<0.01; ***p<0.001	

A2. Free-choice trials

	Dependent variable:	
	congruent choices	RT
	(1)	(2)
congruency		5.787
		t = 3.460***
prime_HighNo	0.013	-1.750
	t = 0.326	t = -0.742
prime_LowNo	-0.116	-4.600
	t = -2.905**	t = -1.960
congruency:prime_HighNo		-0.072
		t = -0.030
congruency:prime_LowNo		-11.065
		t = -4.659***
Constant	0.354	567.476
	t = 1.644	t = 24.974***
Observations	7,029	7,029
Log Likelihood	-4,374.914	-43,955.400
Akaike Inf. Crit.	8,757.829	87,926.800
Bayesian Inf. Crit.	8,785.260	87,981.660
Note:	*p<0.05; **p<0.01; ***p<0.001	

A3. Free-choice trials - with Bins

	Dependent variable:	
	congruent choices	RT
	(1)	(2)
congruency		-2.784
		t = -1.314
prime_HighNo	0.309	-5.416
	t = 3.307***	t = -1.721
BinsBin2	-0.247	68.203
	t = -2.843**	t = 23.421***

BinsBin3	-0.240<br>t = -2.761**	117.743<br>t = 40.519***
BinsBin4	-0.362<br>t = -4.172***	178.824<br>t = 61.673***
BinsBin5	-0.419<br>t = -4.863***	294.982<br>t = 102.343***
prime_LowNo	-0.371<br>t = -4.045***	8.806<br>t = 2.889**
congruency:prime_HighNo		2.025<br>t = 0.639
congruency:BinsBin2		1.356<br>t = 0.465
congruency:BinsBin3		1.952<br>t = 0.670
congruency:BinsBin4		1.099<br>t = 0.379
congruency:BinsBin5		5.540<br>t = 1.919
prime_HighNo:BinsBin2	-0.121<br>t = -0.934	8.431<br>t = 1.925
prime_HighNo:BinsBin3	-0.313<br>t = -2.413*	7.550<br>t = 1.730
prime_HighNo:BinsBin4	-0.586<br>t = -4.538***	0.208<br>t = 0.048
prime_HighNo:BinsBin5	-0.443<br>t = -3.434***	-2.222<br>t = -0.515
congruency:prime_LowNo		-10.040<br>t = -3.279**
BinsBin2:prime_LowNo	0.180<br>t = 1.402	-10.650<br>t = -2.490*
BinsBin3:prime_LowNo	0.245<br>t = 1.923	-12.184<br>t = -2.861**
BinsBin4:prime_LowNo	0.432<br>t = 3.362***	-12.136<br>t = -2.831**
BinsBin5:prime_LowNo	0.406<br>t = 3.190**	-26.122<br>t = -6.146***
congruency:prime_HighNo:BinsBin2		-3.728<br>t = -0.852
congruency:prime_HighNo:BinsBin3		-6.693<br>t = -1.533
congruency:prime_HighNo:BinsBin4		-6.672<br>t = -1.539
congruency:prime_HighNo:BinsBin5		-17.844<br>t = -4.129***

congruency:BinsBin2:prime_LowNo		1.778<br>t = 0.416
congruency:BinsBin3:prime_LowNo		1.354<br>t = 0.318
congruency:BinsBin4:prime_LowNo		2.109<br>t = 0.492
congruency:BinsBin5:prime_LowNo		10.430<br>t = 2.452*
Constant	0.612<br>t = 2.717**	433.821<br>t = 18.734***
Observations	7,029	7,029
Log Likelihood	-4,346.256	-39,937.890
Akaike Inf. Crit.	8,724.512	79,939.790
Bayesian Inf. Crit.	8,834.237	80,159.240
Note:		*p<0.05; **p<0.01; ***p<0.001

A4. Distributional analysis: choices

	Dependent variable:				
	congruency				
	Bin1	Bin2	Bin3	Bin4	Bin5
prime_HighNo	0.065<br>t = 3.445***	0.042<br>t = 2.184*	-0.003<br>t = -0.155	-0.060<br>t = -3.066**	-0.029<br>t = -1.486
prime_LowNo	-0.079<br>t = -4.180***	-0.042<br>t = -2.200*	-0.026<br>t = -1.338	0.013<br>t = 0.672	0.008<br>t = 0.394
Constant	0.627<br>t = 12.408***	0.575<br>t = 11.607***	0.576<br>t = 12.808***	0.551<br>t = 13.178***	0.538<br>t = 13.568***
Observations	1,395	1,401	1,404	1,403	1,426
Log Likelihood	-870.442	-922.167	-939.021	-957.952	-992.143
Akaike Inf.Crit	1,750.885	1,854.334	1,888.042	1,925.904	1,994.287
BayesianInf.Crit	1,777.088	1,880.558	1,914.277	1,952.135	2,020.600
Note:					*p<0.05; **p<0.01; ***p<0.001

A5. Distributional analysis: free-choice RT

	Dependent variable: RT				
	Bin1	Bin2	Bin3	Bin4	Bin5
congruency	1.004<br>t = 0.641	1.398<br>t = 1.097	0.633<br>t = 0.446	-3.215<br>t = -1.696	-1.343<br>t = -0.449
prime_HighNo	-2.039<br>t = -0.924	2.139<br>t = 1.194	1.622<br>t = 0.808	-4.596<br>t = -1.715	-10.105<br>t = -2.363*

prime_LowNo	5.436<br>t = 2.540*	-1.464<br>t = -0.829	-3.116<br>t = -1.579	-4.013<br>t = -1.481	-14.352<br>t = -3.352***
congruency:prime_HighNo	-0.665<br>t = -0.291	-3.911<br>t = -2.127*	-5.411<br>t = -2.623**	-3.271<br>t = -1.213	-12.971<br>t = -3.009**
congruency:prime_LowNo	-7.470<br>t = -3.409***	-7.704<br>t = -4.276***	-8.512<br>t = -4.236***	-9.462<br>t = -3.475***	-0.860<br>t = -0.201
Constant	434.685<br>t = 23.557***	501.901<br>t = 25.802***	551.722<br>t = 25.702***	612.532<br>t = 23.683***	729.066<br>t = 22.123***
Observations	1,395	1,401	1,404	1,403	1,426
Log Likelihood	-7,442.411	-7,250.316	-7,435.216	-7,848.814	-8,648.503
Akaike Inf. Crit.	14,900.820	14,516.630	14,886.430	15,713.630	17,313.010
Bayesian Inf. Crit.	14,942.750	14,558.590	14,928.410	15,755.600	17,355.110

Note: *p<0.05; **p<0.01; ***p<0.001

B. Experiment 2.

B1. Forced-choice trials

	Dependent variable: RT (1)	Dependent variable: Accuracy (2)
congruency	8.478<br>t = 3.456***	-0.262<br>t = -2.731**
target_HighNo	-45.780<br>t = -12.623***	0.461<br>t = 3.069**
target_LowNo	-32.883<br>t = -9.026***	-0.049<br>t = -0.364
congruency:target_HighNo	6.811<br>t = 1.878	0.041<br>t = 0.274
congruency:target_LowNo	-5.129<br>t = -1.408	-0.267<br>t = -1.973*
Constant	561.119<br>t = 22.175***	3.677<br>t = 17.941***
Observations	4,920	4,834
Log Likelihood	-32,082.330	-730.772
Akaike Inf. Crit.	64,182.660	1,477.545
Bayesian Inf. Crit.	64,241.170	1,529.412

Note: #p<0.1, *p<0.05; **p<0.01; ***p<0.001

B2. Free-choice trials

	Dependent variable: congruent choices (1)	Dependent variable: RT (2)

	(1)	(2)
congruency		4.309<br>t = 4.008***
prime_HighNo	0.122<br>t = 3.233**	-9.851<br>t = -6.205***
prime_LowNo	-0.130<br>t = -3.463***	-11.485<br>t = -7.233***
congruency:prime_HighNo		2.153<br>t = 1.315
congruency:prime_LowNo		0.054<br>t = 0.033
Constant	0.031<br>t = 0.654	521.563<br>t = 29.321***
Observations	6,982	6,982
Log Likelihood	-4,819.392	-40,934.300
Akaike Inf. Crit.	9,646.784	81,886.600
Bayesian Inf. Crit.	9,674.189	81,948.260
Note:	*p<0.05; **p<0.01; ***p<0.001	

B3. Free-choice trials - with Bins

	Dependent variable:	
	congruent choices<br>(1)	RT<br>(2)
congruency		2.932<br>t = 2.384*
prime_HighNo	0.255<br>t = 2.939**	-9.659<br>t = -5.262***
BinsBin2	-0.324<br>t = -3.996***	62.655<br>t = 36.561***
BinsBin3	-0.389<br>t = -4.802***	102.328<br>t = 59.683***
BinsBin4	-0.407<br>t = -5.017***	145.863<br>t = 85.012***
BinsBin5	-0.321<br>t = -3.980***	214.652<br>t = 125.795***
prime_LowNo	-0.131<br>t = -1.536	-13.862<br>t = -7.690***
congruency:prime_HighNo		6.538<br>t = 3.542***
congruency:BinsBin2		-4.019<br>t = -2.342*
congruency:BinsBin3		-3.682<br>t = -2.142*

congruency:BinsBin4		-3.276<br>t = -1.902
congruency:BinsBin5		-0.547<br>t = -0.319
prime_HighNo:BinsBin2	-0.218<br>t = -1.800	-1.059<br>t = -0.414
prime_HighNo:BinsBin3	-0.173<br>t = -1.437	0.972<br>t = 0.382
prime_HighNo:BinsBin4	-0.192<br>t = -1.572	0.186<br>t = 0.072
prime_HighNo:BinsBin5	-0.078<br>t = -0.650	1.555<br>t = 0.610
congruency:prime_LowNo		-2.867<br>t = -1.581
BinsBin2:prime_LowNo	0.047<br>t = 0.389	6.746<br>t = 2.659**
BinsBin3:prime_LowNo	-0.069<br>t = -0.570	3.616<br>t = 1.416
BinsBin4:prime_LowNo	0.013<br>t = 0.105	2.242<br>t = 0.887
BinsBin5:prime_LowNo	0.007<br>t = 0.056	-1.710<br>t = -0.677
congruency:prime_HighNo:BinsBin2		-5.331<br>t = -2.085*
congruency:prime_HighNo:BinsBin3		-5.267<br>t = -2.072*
congruency:prime_HighNo:BinsBin4		-7.486<br>t = -2.905**
congruency:prime_HighNo:BinsBin5		-6.483<br>t = -2.543*
congruency:BinsBin2:prime_LowNo		3.775<br>t = 1.488
congruency:BinsBin3:prime_LowNo		2.300<br>t = 0.900
congruency:BinsBin4:prime_LowNo		3.014<br>t = 1.193
congruency:BinsBin5:prime_LowNo		4.652<br>t = 1.842
Constant	0.321<br>t = 4.518***	416.056<br>t = 23.277***
Observations	6,982	6,982
Log Likelihood	-4,800.624	-36,074.410
Akaike Inf. Crit.	9,633.248	72,212.820
Bayesian Inf. Crit.	9,742.865	72,432.050

Note: *p<0.05; **p<0.01; ***p<0.001

B4. Distributional analysis: choices

	Dependent variable: congruency				
	Bin1	Bin2	Bin3	Bin4	Bin5
prime_HighNo	0.059	0.007	0.020	0.015	0.043
	t = 2.900**	t = 0.342	t = 0.962	t = 0.687	t = 2.074*
prime_LowNo	-0.029	-0.019	-0.049	-0.028	-0.030
	t = -1.435	t = -0.907	t = -2.305*	t = -1.346	t = -1.447
Constant	0.578	0.499	0.483	0.479	0.500
	t = 20.011***	t = 21.687***	t = 30.090***	t = 28.402***	t = 29.693***
Observations	1,387	1,388	1,398	1,389	1,420
Log Likelihood	-981.100	-1,010.150	-1,020.738	-1,015.120	-1,037.206
Akaike Inf. Crit.	1,972.201	2,030.300	2,051.476	2,040.240	2,084.412
Bayesian Inf. Crit.	1,998.375	2,056.478	2,077.690	2,066.421	2,110.704

Note: *p<0.05; **p<0.01; ***p<0.001

B5. Distributional analysis: free-choice RT

	Dependent variable: RT				
	Bin1	Bin2	Bin3	Bin4	Bin5
congruency	7.151	-0.579	-1.042	-0.515	1.360
	t = 5.831***	t = -0.719	t = -1.176	t = -0.536	t = 0.919
prime_HighNo	-10.135	-11.448	-8.702	-9.836	-8.705
	t = -5.642***	t = -9.670***	t = -6.751***	t = -6.850***	t = -3.977***
prime_LowNo	-13.588	-6.524	-10.277	-11.150	-14.619
	t = -7.706***	t = -5.499***	t = -7.745***	t = -7.928***	t = -6.677***
congruency	3.063	-0.894	0.490	-0.134	1.820
:prime_HighNo	t = 1.658	t = -0.731	t = 0.363	t = -0.091	t = 0.818
congruency	2.245	2.065	-0.122	-0.586	0.912
:prime_LowNo	t = 1.237	t = 1.707	t = -0.088	t = -0.408	t = 0.409
Constant	416.164	478.569	518.297	562.003	631.297
	t = 30.722***	t = 30.606***	t = 29.879***	t = 28.733***	t = 26.074***
Observations	1,387	1,388	1,398	1,389	1,420
Log Likelihood	-7,153.973	-6,631.957	-6,821.885	-6,888.771	-7,663.597
Akaike Inf. Crit.	14,323.950	13,279.920	13,659.770	13,793.540	15,343.190
Bayesian Inf. Crit.	14,365.830	13,321.800	13,701.710	13,835.430	15,385.260

Note: *p<0.05; **p<0.01; ***p<0.001

C. Analysing order effects

Below, we report the analyses of free-choice trials with order (Reward session first: +1, reward session second: -1) as a factor along with the factors mentioned in the main text. Only relevant interactions involving "order" are included here. For other main effects and interactions, please refer to the main text. The full model outputs involving "order" are given below after the description of the results.

Experiment 1

Choices. There was a significant interaction between order and HighNo condition, $\beta = -0.09$, $t = -2.22$, $p = 0.026$. The reduction in priming effects for the low-reward condition compared to the no-reward condition was greater when the reward session was administered first. The interaction between LowNo and order was not significant (p = 0.381).

RT. There was a significant 3-way interaction between congruency, HighNo and order, $\beta = 6.45$, $t = 2.70$, $p = 0.007$. The reduction in priming effects for the high-reward condition compared to no-reward condition was greater when the reward session was administered second. The 3-way interaction between congruency, LowNo and order was not significant (p = 0.749).

	Dependent variable:	
	congruent choices (1)	RT (2)
congruency		5.581 t = 3.333***
prime_HighNo	0.030 t = 0.739	0.608 t = 0.259
order_coded	0.070 t = 0.324	5.942 t = 0.254
prime_LowNo	-0.123 t = -3.036**	-3.310 t = -1.419
congruency:prime_HighNo		-1.138 t = -0.476
congruency:order_coded		2.298 t = 1.373
prime_HighNo:order_coded	-0.090 t = -2.220*	-16.842 t = -7.176***
congruency:prime_LowNo		-12.060 t = -5.111***
order_coded:prime_LowNo	0.036 t = 0.876	-11.248 t = -4.821***
congruency:prime_HighNo:order_coded		6.451 t = 2.702**
congruency:order_coded:prime_LowNo		-0.755

		t = -0.320
Constant	0.346<br>t = 1.604	567.064<br>t = 24.272***
Observations	7,029	7,029
Log Likelihood	-4,372.138	-43,831.370
Akaike Inf. Crit.	8,758.276	87,690.750
Bayesian Inf. Crit.	8,806.280	87,786.760
Note:	*p<0.05; **p<0.01; ***p<0.001	

Experiment 2

Choices. There was a significant interaction between order and LowNo, $\beta = -0.08$, $t = -2.07$, $p = 0.038$ and a marginally significant interaction between order and HighNo, $\beta = 0.06$, $t = 1.75$, $p = 0.081$. The increase in congruent choices for high-reward condition compared to no-reward was greater when reward session was presented first. Similarly, the decrease in congruent choices for low-reward condition compared to no-reward was also greater when reward session was presented first.

RT. There were no significant 3-way interactions between congruency, prime conditions (LowNo or HighNo) and order ($p > 0.7$) suggesting that the reward-mediated priming effects were not modulated by the order in which reward and control sessions were presented.

	Dependent variable:	
	congruent choices<br>(1)	RT<br>(2)
congruency		4.208<br>t = 3.946***
prime_HighNo	0.122<br>t = 3.236**	-9.737<br>t = -6.180***
order_coded	0.048<br>t = 1.041	-13.740<br>t = -0.756
prime_LowNo	-0.130<br>t = -3.465***	-11.554<br>t = -7.338***
congruency:prime_HighNo		1.412<br>t = 0.869
congruency:order_coded		2.091<br>t = 1.961*
prime_HighNo:order_coded	0.066<br>t = 1.746	-9.865<br>t = -6.261***
congruency:prime_LowNo		0.353<br>t = 0.218
order_coded:prime_LowNo	-0.078<br>t = -2.073*	-4.920<br>t = -3.124**

```
congruency:prime_HighNo:order_coded                                0.290
                                                                 t = 0.178

congruency:order_coded:prime_LowNo                                 0.574
                                                                 t = 0.355

Constant                                          0.031          521.571
                                                t = 0.678    t = 28.716***

---------------------------------------------------------------------------
Observations                                      6,982           6,982
Log Likelihood                                 -4,816.480     -40,860.530
Akaike Inf. Crit.                               9,646.960      81,751.060
Bayesian Inf. Crit.                             9,694.917      81,853.830
===========================================================================
Note:                                        *p<0.05; **p<0.01; ***p<0.001
```

D. Response type analysis

In one version of the task in Experiment 1, participants received high-reward on seeing target 1 and low-reward for target 2 on forced-choice trials. They were asked to press A for target 1 and L for target 2. The table below gives all possible prime-response combinations on free-choice trials and the reward-levels for both prime-type and response-type (for Experiment 1, as an example). The analysis was done based on "response-type" in both experiments to examine if mere response-related associations lead to the observed effects in a prime-independent manner.

Prime	Prime-type	Response	Response-type
1	High-reward	A	High-reward
2	Low-reward	A	High-reward
1	High-reward	L	Low-reward
2	Low-reward	L	Low-reward
